# Testicular heterochrony in *vgll3*-mediated maturation age in Atlantic salmon

**DOI:** 10.1093/g3journal/jkaf196

**Published:** 2025-08-21

**Authors:** Ehsan Pashay Ahi, Jukka-Pekka Verta, Johanna Kurko, Annukka Ruokolainen, Paul Vincent Debes, Craig R Primmer

**Affiliations:** Organismal and Evolutionary Biology Research Programme, Faculty of Biological and Environmental Sciences, University of Helsinki, Helsinki 00014, Finland; Organismal and Evolutionary Biology Research Programme, Faculty of Biological and Environmental Sciences, University of Helsinki, Helsinki 00014, Finland; Faculty of Biosciences and Aquaculture, Nord University, Bodø 8026, Norway; Organismal and Evolutionary Biology Research Programme, Faculty of Biological and Environmental Sciences, University of Helsinki, Helsinki 00014, Finland; Organismal and Evolutionary Biology Research Programme, Faculty of Biological and Environmental Sciences, University of Helsinki, Helsinki 00014, Finland; Organismal and Evolutionary Biology Research Programme, Faculty of Biological and Environmental Sciences, University of Helsinki, Helsinki 00014, Finland; Department of Aquaculture and Fish Biology, Hólar University, Hólar 551, Iceland; Organismal and Evolutionary Biology Research Programme, Faculty of Biological and Environmental Sciences, University of Helsinki, Helsinki 00014, Finland

**Keywords:** heterochrony, gene expression, maturation timing, vgll3-Hippo pathway, testis, Atlantic salmon

## Abstract

Heterochrony, or shifts in developmental timing, drives phenotypic diversity within and between species and shapes life history traits that can be selected for in changing environments, which in turn promotes population resilience. Mutations in heterochronic genes that regulate these processes can induce stable timing shifts, impacting important life history traits such as pubertal timing. Age at maturity is a key adaptive trait across species, with *vestigial-like family member 3* (*vgll3*), a Hippo pathway cofactor, as a main determinant in Atlantic salmon. Recent studies show that *early* (E) and *late* (L) *vgll3* alleles affect reproductive gene expression in salmon, reinforcing its role in regulating developmental timing. This study examines whether *vgll3* influences testicular heterochrony in Atlantic salmon by analyzing gene expression related to the Hippo pathway. We observed heterochronic divergence in Hippo pathway gene transcription, indicating accelerated changes linked to spermatogenesis in vgll3*EE individuals. Our results position *vgll3* as a heterochronic gene with a key role in regulating developmental timing in salmon.

## Graphical Abstract

**Graphical Abstract:**
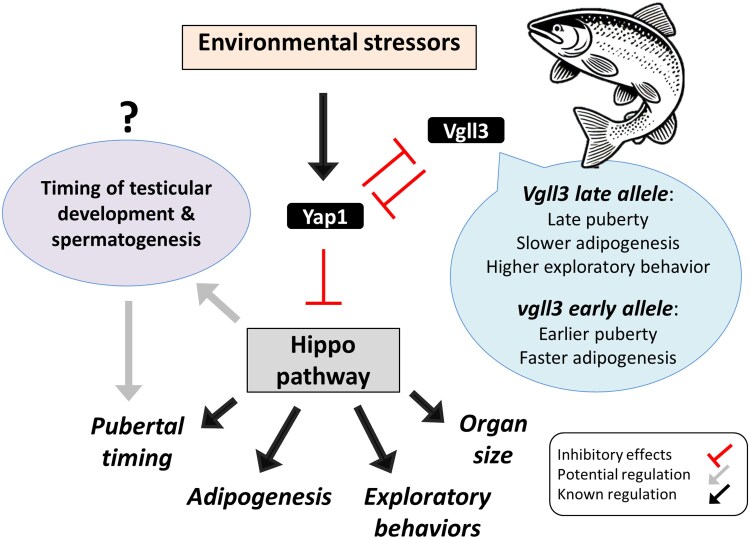

## Introduction

Changes in the timing of developmental events, known as heterochrony, contribute to the phenotypic differences observed within and between species (Rougvie 2001; Garcia-Ojalvo and Bulut-Karslioglu 2023). Heterochrony involves shifts in the onset, completion, or rate of developmental events relative to an ancestor. Understanding these timing mechanisms is essential for grasping their evolutionary impact (Smith 2003). Throughout an individual's life, environmental stressors may also influence these heterochronic events, a process known as heterokairy, adding another layer of phenotypic diversity at the individual level (Rundle and Spicer 2016). This becomes especially likely if the molecular signals driving heterochronic events are also responsive to environmental changes (Dubansky 2018; Spicer et al. 2018). Heterochronic processes give rise to life history trait variation and may sometimes be the targets of selection even contributing to major evolutionary processes such as vertebrate evolution (McNamara 2012). Alternative life history strategies arising from various traits significantly enhance population diversity, supporting adaptive capacity and resilience (Brommer 2000). Mutations in genes regulating heterochronic processes, so-called “heterochronic genes,” if advantageous, could drive stable shifts within populations, potentially becoming key sources of diversity (Rougvie 2001). A classic example of heterochronic genes is a highly conserved family of RING finger/B-box proteins known as *Lin* genes (Ambros and Horvitz 1984). These genes regulate the timing of developmental events across various organisms such as pubertal timing (age at maturity) (Rougvie 2001).

Age at maturity is a key adaptive life history trait in many organisms, but in humans, shifts in maturation timing, leading to early or delayed puberty, can introduce various physical and psychological challenges (Hartge 2009; Day et al. 2017; Leka-Emiri et al. 2017). One well-studied example of a heterochronic gene affecting age at maturity is the *Lin28a/b* gene, a highly conserved factor influencing body size, pubertal timing, and metabolism from worms to humans (Ong et al. 2009; Zhu et al. 2010). The impact of *Lin28a/b* on pubertal timing appears sex specific, primarily affecting males, and is linked to metabolic changes related to adiposity (Zhu et al. 2010; Faas et al. 2013; Ouchi et al. 2014; Corre et al. 2016). A recent discovery has shown that *Lin28a/b* can act partly independently of *let-7* by directly inhibiting the Hippo pathway, which is involved in sexual maturation and organ size control, through the induction of *YAP1* expression, a negative regulator of the pathway (Luo et al. 2021; Zou et al. 2022). This finding could explain the role of *Lin28a/b* in all the processes mentioned earlier, as the Hippo pathway is a key regulator of body size (Pan 2007), adipogenesis (Ardestani et al. 2018; Shen et al. 2022), and sexual maturation via the hypothalamus–pituitary–gonadal (HPG) axis (Sen Sharma et al. 2019; Lalonde-Larue et al. 2022; Ahi et al. 2025b).

**Fig. 1.**
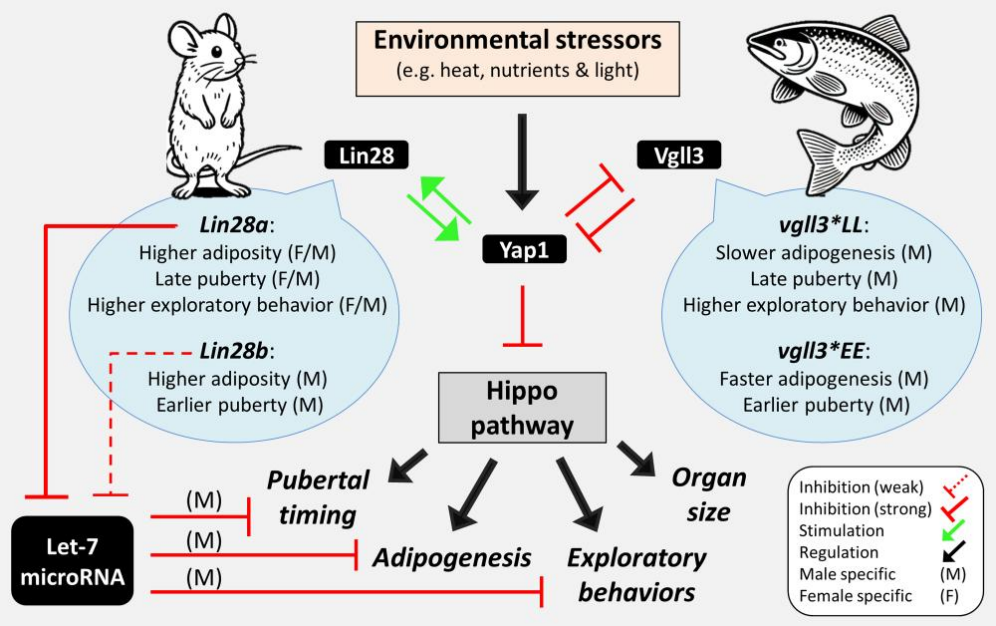
Functional similarities between *Lin28* in mice and *vgll3* in Atlantic salmon. *Lin28* is a well-known heterochronic gene that regulates pubertal timing across various species and, in mammals, is also associated with adiposity and behavioral effects (i.e. exploratory behavior and boldness). The functional similarities between *Lin28* and *vgll3* in these processes are thought to occur through differential activation of the Hippo pathway (via Yap1 regulation). However, the sex specificity of *Lin28* in mammals appears distinct from that of *vgll3* in salmon, potentially due to *Lin28a/b*'s additional role in regulating the biogenesis of *let-7* (a highly conserved microRNA playing a role in developmental heterochrony). *let-7* influences pubertal timing, adipogenesis, and behavior in a sex-specific manner, with effects more pronounced in males. Since *Lin28a/b* is a stronger suppressor of *let-7*, its induction could override *let-7*'s sex-specific effects in these processes. It is currently unknown whether the male-specific effects of *vgll3* is Hippo pathway dependent or through other molecular players such as *let-7*. However, these similarities indicate a potential role of *vgll3* in developmental heterochrony in Atlantic salmon.

In Atlantic salmon, the gene *vestigial-like family member 3* (*vgll3*) is a primary genetic determinant of maturation timing, accounting for over 39% of the variation in age at maturity within natural populations (Barson et al. 2015; Czorlich et al. 2018). Although *vgll3* has not been categorized as a heterochronic gene, its function in salmon physiology closely mirrors the roles of *Lin28a/b*, showing sex-specific effects on age at maturity (Barson et al. 2015; Czorlich et al. 2018; Åsheim et al. 2024; Miettinen et al. 2024), influencing adiposity and seasonal energy storage (House et al. 2023, 2025; Ahi et al. 2024b), and regulating the Hippo pathway by inhibiting Yes-associated protein 1 (YAP1) (Hori et al. 2020; Kurko et al. 2020) (Fig. 1). Interestingly, like *Lin28a/b*, these functions of *vgll3* appear to be evolutionarily conserved. For example, *vgll3* has been linked to age at maturity in humans (Perry et al. 2014; Cousminer et al. 2016), regulation of adipogenesis in mice (Halperin et al. 2013), and sex-specific functions in humans, e.g. sex-biased pathogenesis of autoimmune diseases (Liang et al. 2016; Plazyo et al. 2025). Our recent investigations involving male Atlantic salmon have revealed strong correlations between *vgll3* alleles E and L linked to *early* and *late* maturation, respectively, and the expression patterns of important reproductive genes along the HPG axis (Ahi et al. 2022, 2023; Verta et al. 2024).

Heterochronic shifts in transcription are not uniformly reflected across all organs (Garcia-Ojalvo and Bulut-Karslioglu 2023). In mammals, for example, the brain exhibits minimal heterochronic divergence in developmental events, while the testis shows the most pronounced heterochrony across species (Cardoso-Moreira et al. 2019). Particularly, within the testis, transcriptional changes associated with different stages of spermatogenesis display the most significant heterochronic divergence between mammalian species (Cardoso-Moreira et al. 2019). Recent studies have examined the potential functions of *vgll3* in the testis of Atlantic salmon, ranging from isoform-specific (Verta et al. 2020) and cell-specific regulatory roles (Kjærner-Semb et al. 2018) to broader interactions with other pathways involved in testicular development, such as the TGF-β and Wnt signaling pathways (Verta et al. 2024). Beyond these, further pathway-specific molecular investigations would provide deeper insights into the role of the Hippo pathway in testicular maturation.

In this study, we aimed to investigate whether the effects of *vgll3* on testis involve heterochronic shifts in the transcription of its downstream molecular targets, i.e. known components of the Hippo pathway as well as their associated interacting partners and signaling molecules. We assessed the testicular expression of these genes in the immature and mature Atlantic salmon, with distinct *early* (E) or *late* (L) maturing *vgll3* genotypes. Through a comparative analysis of maturity status and *vgll3* genotype, we employed methodologies centered on pathways and regulatory networks. The results of our study could be ecologically significant, as the Hippo pathway is emerging as a key sensor of environmental changes, such as temperature and food availability (Shu et al. 2019; Luo et al. 2020; Ahi et al. 2025a). The identification of Hippo-*vgll3*-mediated heterochronic shifts suggests that these shifts may also be influenced by environmental stressors, potentially leading to variation in the timing of related developmental events within an individual's life—a phenomenon known as heterokairy (Rundle and Spicer 2016). This finding positions *vgll3* not only as a conserved heterochronic gene but also as a potential heterokairic gene, relevant for studies of adaptation linked to heterochrony within and between populations (see Box 1 for definitions of key terms).

Box 1. Glossary of key terms **Heterochrony:** Shifts in the timing, rate, or duration of developmental events, arising through evolution or induced by genetic perturbation (e.g. mutagenesis), that alter phenotype within and across species.

**Heterokairy:** Environmentally induced variation in the timing of developmental processes within individuals, often in response to stressors.

**Heterochronic gene:** A gene that regulates developmental timing. Mutations in such genes can alter traits such as age at maturity.

***vgll3* (*vestigial-like family member 3*):** A gene involved in developmental regulation and a major determinant of age at maturity in Atlantic salmon, with early (E) and late (L) alleles.

**Hippo pathway:** A signaling pathway that controls organ size, cell proliferation, and development and acts as a sensor of environmental cues.

**YAP1 (Yes-associated protein 1):** A transcription coactivator and key effector of the Hippo pathway that promotes growth and sexual maturation. It has a function opposing *vgll3*, with both proteins competing for binding to TEAD (TEA Domain Family) transcription factors.

**HPG axis (hypothalamus–pituitary–gonadal axis):** The hormonal system that governs reproductive development and function in vertebrates.

**Life history traits:** Biological characteristics of an organism such as growth rate, age at maturity, and reproductive output that influence an organism’s reproductive success and/or survival, i.e. fitness.

**Developmental timing:** The chronological schedule of key developmental events in an organism’s life, regulated by both genetic and environmental factors.

## Materials and methods

### Fish material and tissue sampling

The individuals involved in this study were sourced from the same population (Oulujoki) and cohort as previously detailed in Verta et al. (2020), which provided access to individually PIT (Passive Integrated Transponder)-tagged individuals with known *vgll3* genotypes (refer to Verta et al. (2020) for more information on breeding and rearing). All individuals were genotyped for *vgll3* and PIT tagging allowed for genotype identification during later sampling. At each stage, individual PIT tags were scanned and their genotypes were identified, and the required number of vgll3*EE and vgll3*LL individuals were selected accordingly. For the purposes of this study, male individuals were collected at the age range of 1.5 to 2 years postfertilization (Debes et al. 2020). In total, 24 individuals were sampled: 4 biological replicates for each of the 2 *vgll3* genotypes (EE and LL) across 3 maturation stages (4 × 2 × 3 = 24). The euthanization process involved the administration of an overdose of MS222, after which the entire testes from each male were collected at 1 of 3 time points, representing distinct stages of maturation development (as described below). Subsequently, these tissue samples were snap frozen with liquid nitrogen and stored at −80 °C until further processing.

*Immature 1*-stage individuals were gathered during the late spring period (May 5 to 21) and exhibited an average mass of 17.5 g, ranging from 10.2 to 27.4 g. Their average length measured 12.1 cm, with individual lengths falling within the range of 9.1 to 13.6 cm. These individuals did not display any noticeable signs of testicular maturation, as indicated by a gonadosomatic index (GSI) value of 0.

*Immature 2*-stage individuals were sampled in the summer season (July 4 to 17). They had an average mass of 33.7 g, ranging from 21.1 to 76.6 g, and an average length of 17.3 cm, falling within the range of 14 to 18.5 cm. Some of these individuals exhibited initial indications of phenotypic maturation processes, as reflected by GSI values ranging from 0.0 to 0.3.

*Mature*-stage individuals were collected close to the anticipated spawning period in early autumn (October 1 to 15). These individuals had an average mass of 82.7 g, with a range of 60.3 to 120.6 g, and an average length of 20.8 cm, ranging from 16.4 to 22.7 cm. All individuals in this group displayed well-developed testes, indicative of maturity, with a GSI exceeding 3.

### RNA extraction

RNA extraction and sample preparation for NanoString analysis were performed in a single batch after all sampling stages were completed. RNA was extracted from 24 testis samples using a NucleoSpin RNA kit (Macherey-Nagel GmbH & Co. KG). The collected samples were processed by placing them in tubes containing 1.4 mm ceramic beads from Omni International, along with Buffer RA1 and DDT/dithiothreitol (350 µl of RA1 and 3.5 µl of 1 M DDT). Homogenization was carried out using the Bead Ruptor Elite (Omni International) at a frequency of 30 Hz for a total of 2 min, involving 6 cycles of 20 s each. The subsequent RNA extraction procedures followed the manufacturer's guidelines, including an integrated DNase stage to eliminate any remaining genomic DNA. After completion of the process, RNA from each individual sample was eluted using 50 µl of nuclease-free water. RNA quantity was assessed using the NanoDrop ND-1000 instrument (Thermo Scientific, Wilmington, DE, USA), and RNA quality was evaluated with the 2100 Bioanalyzer system (Agilent Technologies, Santa Clara, CA, USA). All samples had a RNA integrity number (RIN) exceeding 7, and 100 ng of extracted RNA from each isolation was used for the hybridization step in the NanoString panel. The extracted RNA samples were randomly distributed across the NanoString plate to minimize potential batch effects related to run order or sample grouping.

### NanoString nCounter panel

The NanoString nCounter technology is a versatile method for simultaneously assessing the expression of a great number of RNA molecules in a dependable and reproducible manner (Goytain and Ng 2020). This technology offers valuable advantages for ecological and evolutionary research, as it requires minimal RNA input and can handle lower-quality RNA, making it a more accessible option compared to RNA-seq. Importantly, it does not involve an amplification step and can detect even extremely low levels of RNA expression. In this study, an expanded NanoString panel of probes was used, building upon a previous investigation (Kurko et al. 2020) that initially focused on gene expression related to age at maturity in Atlantic salmon. This updated panel includes >100 additional genes, with a specific focus on a comprehensive collection of Hippo pathway components and other genes directly linked to the Hippo pathway. The selection process involved a combination of literature review and the use of tools such as Ingenuity Pathway Analysis (IPA) from Qiagen, along with other web-based tools and databases (Kurko et al. 2020). Notably, the panel comprises probes targeting genes associated with age at maturity in Atlantic salmon, such as *vgll3a* on chromosome 25 and *six6a* on chromosome 9, along with their corresponding paralogs, *vgll3b* on chromosome 21 and *six6b* on chromosome 1. Additionally, the panel includes probes for other genes functionally connected to sexual maturation, including those within the HPG axis. Given that most candidate genes have multiple paralogs due to the duplicated Atlantic salmon genome (Lien et al. 2016), the paralogs of each gene of interest were integrated and identified through resources such as SalmoBase (http://salmobase.org/) and the NCBI RefSeq databases. Further information regarding gene/paralog selection and nomenclature can be found in Kurko et al. (2020). The gene accession numbers, symbols, full names, and functional classifications are provided in Supplementary File 1. The analysis of mRNA expression levels for these candidate genes involved the use of the NanoString nCounter Analysis technology (NanoString Technologies, Seattle, WA, USA). Probes designed for each gene paralog, targeting all known transcript variants, were formulated using reference sequences from the NCBI RefSeq database. However, designing paralog-specific probes was not feasible for some genes due to high sequence similarity between paralogs. In practice, the execution involved the use of the nCounter Custom CodeSet for probes and the nCounter Master kit (NanoString Technologies). The RNA from each sample was denatured, followed by an overnight hybridization with the probes. Purification and image scanning were performed on the following day.

### Data analysis

Raw NanoString counts were normalized using a 2-step procedure following NanoString's recommended guidelines. First, positive control probes were used to adjust for technical variability across samples. Second, reference gene normalization was performed using the geometric mean of 9 housekeeping genes previously validated for stable expression in salmonids. The normalized count for each gene was calculated by dividing raw counts by the geometric mean of the reference genes and scaling to the average geometric mean across all samples. For analyses of immature individuals, all 9 candidate reference genes in the panel (*actb*; *ef1a* paralogs, *ef1aa*, *ef1ab*, and *ef1ac*; *gapdh*; *hprt1*; *prabc2* paralogs, *prabc2a* and *prabc2b*; and *rps20*) were used for data normalization due to their low coefficient of variation (CV) values across the samples. For analyses of mature individuals, one of the candidate reference genes, *actb*, was excluded because it displayed high variation (CV% > 50), and although it is commonly used as a reference gene in many studies, it appeared unsuitable for data normalization in the mature testis of Atlantic salmon. After this selection process, the raw count data obtained from NanoString nCounter mRNA expression was normalized using RNA content normalization factors, which were calculated individually for each sample using the geometric mean count values of the chosen reference genes. Following normalization, a quality control assessment was conducted on the data, and all samples met the predefined threshold using the default criteria of nSolver Analysis Software v4.0 (NanoString Technologies; www.nanostring.com/products/nSolver). This assessment included evaluation of several quality metrics such as imaging quality, binding density (acceptable range: 0.05 to 2.25 spots/μm²), positive control linearity, and background levels estimated from negative controls. Positive controls (synthetic RNA targets of known concentration) were used to assess assay performance, while negative controls (nontarget probes) served to define background signal. For expression-level filtering, a detection threshold was set at the mean of the negative controls plus 2 standard deviations (mean + 2 SD). Genes not exceeding this threshold in at least 1 sample were excluded from downstream analyses. In the data analysis process using the software, the mean of the negative controls was subtracted, and positive control normalization was carried out by utilizing the geometric mean of all positive controls, following the manufacturer's recommendations. To establish a baseline signal threshold, a normalized count value of 20 was set as the background signal. As a result, 140 genes in the panel displayed an average signal below this threshold across the samples, leaving 193 genes for subsequent analyses. Differential expression analysis was performed using the log-linear and negative binomial model (lm.nb function) integrated into NanoString's nSolver Advanced Analysis Module (nS/AAM). Predictive covariates included the maturation status (*Immature 1*, *Immature 2*, *Mature*) and genotypes (vgll3*EE, vgll3*LL), following nS/AAM's recommendations. Differential expression was assessed using pairwise comparisons between genotypes within each maturation stage and between maturation stages within each genotype. This approach was designed to capture biologically meaningful transcriptional differences associated with testis maturation and genotype-specific regulation. Each stage, sampled in a distinct season, was treated as a separate biological condition reflecting the discrete, seasonal nature of spermatogenesis in Atlantic salmon. To account for multiple hypothesis testing, the Benjamini–Yekutieli method (Benjamini and Yekutieli 2001) was applied across all tests, and genes with an adjusted *P* < 0.05 were considered statistically significant (Supplementary File 2). For further exploration, log-transformed expression values were used to calculate pairwise Pearson correlation coefficients (*r*) between the gene expression variation of each candidate gene and GSI variation across all the samples.

We utilized the weighted gene coexpression network analysis (WGCNA) R-package version 1.68 in R-package version 5.2.1 to identify gene coexpression networks (GCNs) following the method by Langfelder and Horvath (2008) to compare the difference between the genotypes. Given our primary focus on comparing alternative *vgll3* genotypes, we utilized all samples, across both maturation statuses and time points within each genotype, as biological replicates to ensure robust statistical power for WGCNA. For this analysis, we included only the 194 genes that met the expression threshold described above. All genes passing this threshold were analyzed within each genotype, regardless of differential expression across genotypes or time points. Log2-transformed, normalized NanoString counts were used as input. To understand sample relationships, we hierarchically clustered samples based on gene expression. The process of building coexpression networks involved (i) calculating gene coexpressions using Pearson correlation coefficients, (ii) creating an adjacency matrix to establish scale-free topology, (iii) computing the topological overlap distance matrix based on the adjacency matrix, (iv) hierarchical clustering of genes using the topological overlap distance, (v) identifying coexpressed gene modules using the cutTreeDynamic function with a minimum module size of 10 genes, (vi) assigning colors to each module and representing module-specific expression profiles through the principal component [module eigengene {ME}], and finally (vii) merging highly similar modules based on ME dissimilarity with a distance threshold of 0.25 to finalize the coexpressed gene modules as described by Singh et al. 2021. Additionally, we conducted conditional coexpression analysis, constructing separate coexpression networks for each *vgll3* genotype to assess the preservation of modules between vgll3*EEvgll3*LL genotypes. We used a softpower of 7 to create an adjacency matrix. To evaluate module preservation, we computed module preservation statistics using WGCNA, following the methodology of Langfelder et al. (2011). A permutation test shuffled genes in the query network and calculated Z-scores. The individual Z-scores from 200 permutations were summarized into a Zsummary statistic.

To gain deeper insights into the coexpression modules identified via WGCNA, we employed WebGestalt (Elizarraras et al. 2024) to evaluate the biological processes linked to the genes in each module. This evaluation was conducted using gene-set enrichment analysis, adjusted for a false discovery rate (FDR) < 0.05, focusing specifically on Gene Ontology/Biological Process (GO/BP) categories. To predict potential gene interactions and identify key interacting hubs, we used differentially expressed genes (DEGs) from each comparison and mapped them to their conserved human orthologs using BioMart from the Ensembl database (Smedley et al. 2009). We chose these orthologs due to their well-established and extensively studied interactome data across vertebrates (Ahi 2025). We then used these orthologs as input for STRING version 12.0, a comprehensive knowledge-based interactome database for vertebrates (Szklarczyk et al. 2023). Predicted gene interactions were based on various factors such as structural similarities, cellular colocalization, biochemical interactions, and coregulation. We maintained a medium confidence level for each interaction or molecular connection prediction, which is the default threshold. The list of Atlantic salmon DEGs, their matched human orthologs identified via Ensembl BioMart, and the input set used in STRING is provided in Supplementary File 2.

## Results

### Testicular gene expression differences between *vgll3* genotypes

**Fig. 2.**
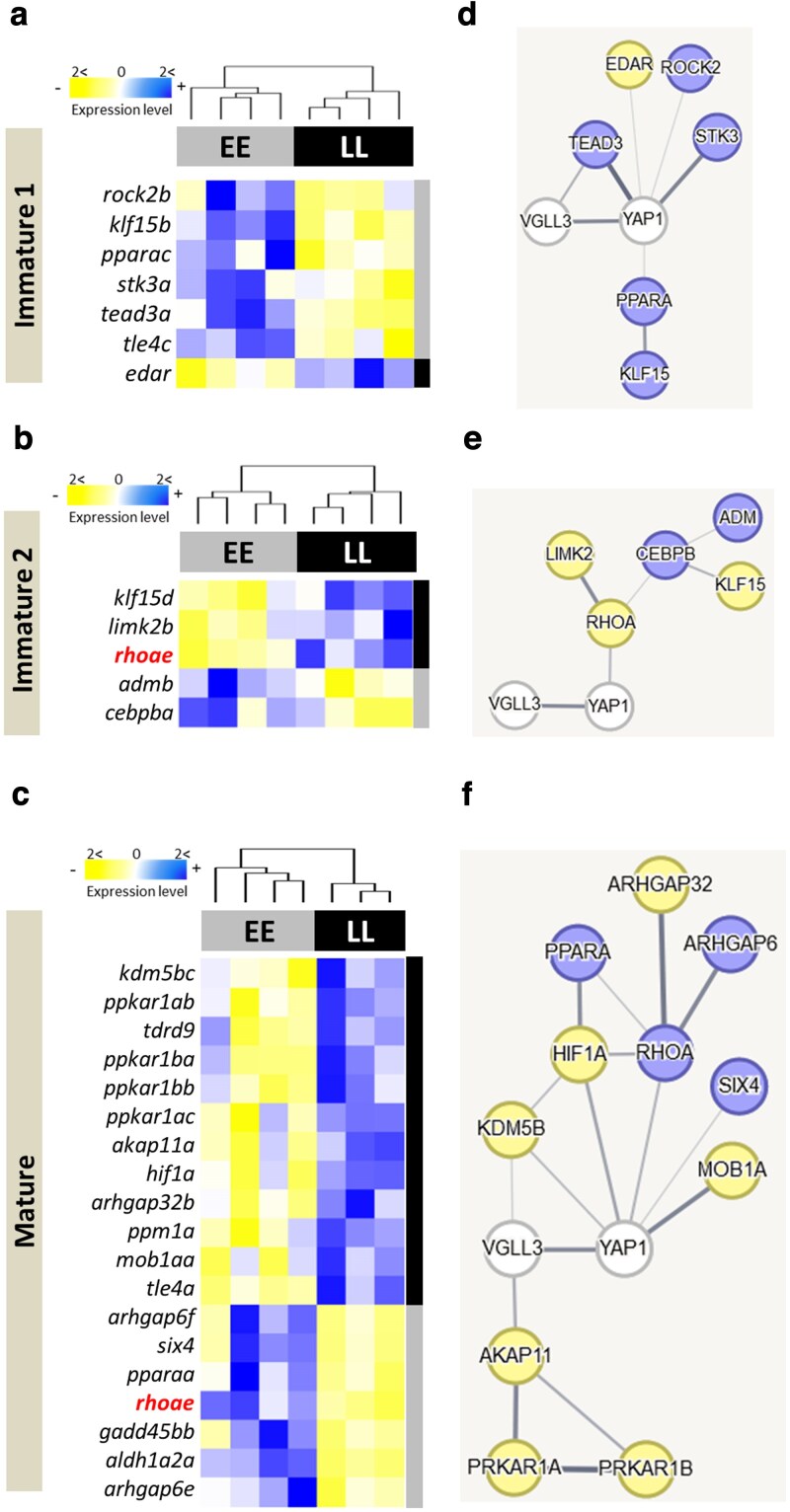
Testicular gene expression differences and predicted interactions between early and late maturation genotypes of *vgll3* in Atlantic salmon. Heatmaps illustrate genes with varying expression levels among alternative homozygous *vgll3* genotypes in Atlantic salmon testis at 3 developmental timepoints a to c). Anticipated molecular connections between these genes are shown using STRING v12 (http://string-db.org/) d to f). The thickness of the lines connecting the genes reflects the relative likelihood of interaction (thicker = more likely).

Initially, we assessed variations in gene expression among the alternative vgll3*EE and vgll3*LL genotypes at the 3 distinct developmental stages described in the methods: *Immature 1*, *Immature 2*, and *Mature* (as shown in Fig. 2). Between the genotypes differentially expressed (DE) genes were exhibited at *Immature 1*, with 7 DE genes, and at *Immature 2*, with 5 DE genes, and 19 DE genes at the *Mature* stage. At *Immature 1*, all the DE genes, except *edar*, displayed higher expression in vgll3*EE genotype individuals (Fig. 2a). Our investigation into molecular interactions revealed that while only 1 gene (*tead3a*) had a direct interaction with *vgll3*, 5 genes exhibited potential direct interactions with *yap1* (*edar*, *pparac*, *rock2b*, *stk3a*, and *tead3a*) (as indicated by connecting lines in Fig. 2b). At *Immature 2*, we observed 2 genes (*admb* and *cebpba*) with higher expression in vgll3*EE genotype individuals, and 3 genes (*klf15d*, *limk2b*, and *rhoae*) showed increased expression in vgll3*LL genotype individuals (as illustrated in Fig. 2c). The predicted interactions indicated that 1 gene (*rhoae*) had a direct molecular interaction with *yap1*, while no gene exhibited direct interactions with *vgll3* (as shown in Fig. 2d). Moreover, the *rhoae* gene, which interacted with *yap1*, formed interacting hubs with 2 other DE genes (*limk2b* and *cebpba*) (Fig. 2d). At the *Mature* stage, we identified 7 genes with higher expression in vgll3*EE genotype individuals and 12 genes that displayed increased expression in vgll3*LL genotype individuals (as presented in Fig. 2e). Our interaction analysis unveiled 5 genes (*kdm5bc*, *hif1a*, *mob1aa*, *rhoae*, and *six4*) with direct interactions with *yap1* and 2 genes (*kdm5bc* and *akap11a*) with direct interactions with *vgll3* (as indicated in Fig. 2f). Among the genes with direct interactions with *yap1*, 2 genes, *rhoae* and *hif1a*, formed interacting hubs, suggesting multiple interactions with other DE genes (Fig. 2f). Ultimately, across all the comparisons, only 1 gene, *rhoae*, displayed differential expression in more than 1 comparison (at *Immature 2* and *Mature*). However, it exhibited lower expression in vgll3*EE at *Immature 2* but higher expression in vgll3*EE genotype individuals at the *Mature* stage.

We analyzed gene expression in relation to gonadal development (GSI) within each genotype to identify genes directly associated with testis growth (Supplementary Fig. 1). In vgll3*LL individuals, the expression level of 38 genes was correlated with GSI; 14 positively and 24 negatively (Supplementary Fig. 1a). In vgll3*EE individuals, the expression level of 19 genes was negatively correlated, but none positively correlated with GSI. Furthermore, the expression of 43 genes was significantly correlated with GSI across both genotypes, including 11 with positive and 31 with negative correlations; additionally, 1 gene showed opposite correlation patterns between the genotypes. The gene with opposite correlation, *lats1a*, exhibited a positive association with GSI in vgll3*LL and a negative association in vgll3*EE. Many of these shared genes ranked among the most strongly GSI correlated (Supplementary Fig. 1b), suggesting a common set of maturation-associated genes.

### Testicular gene expression differences between maturation stages

**Fig. 3.**
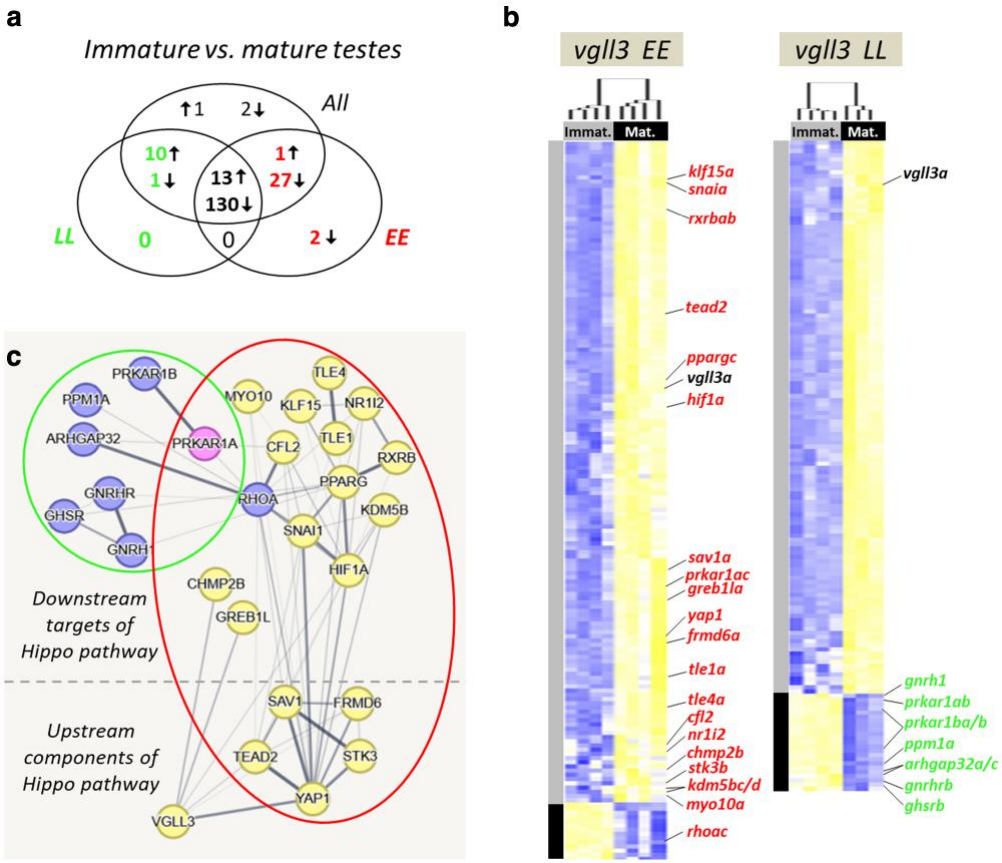
Testicular gene expression differences between immature and mature Atlantic salmon. Venn diagram showing the numbers of DEGs overlapping between the comparisons a). Heatmaps representing DEGs between the immature and mature testes within alternative *vgll3* homozygotes (blue and yellow shadings represent higher and lower expressions, respectively) b). Predicted interactions between the genes in the Venn diagram c). The thickness of the connecting lines between the genes indicates the probability of the interaction (thicker = higher).

To identify differences in gene expression between immature and mature testes, we compared immature individuals collected at both *Immature 1* and *Immature 2* stages with those collected at the *Mature* stage. We identified 187 DE genes between mature and immature individuals when both *vgll3* genotypes were combined. When analyzing each genotype separately, we found 154 DE genes in vgll3*LL genotype individuals and 173 DE genes in vgll3*EE genotype individuals (Fig. 3a). Furthermore, 143 DE genes were found to overlap across all 3 comparisons, indicating differential expression independent of *vgll3* genotype (Fig. 3a). Overall, most DE genes in all 3 comparisons showed average lower expression at the *Mature* stage. A total of 25 genes showed higher expression in the *Mature* stage across the comparisons. Of these, 13 genes were consistently upregulated in mature individuals across all comparisons (Fig. 3a). An additional 10 genes were upregulated in both vgll3*LL and combined comparisons, 1 gene only in the combined comparison (*rhoab*), and 1 gene in both vgll3*EE and combined comparisons (*rhoac*). We observed more genotype-dependent DE genes in vgll3*EE individuals (30 genes, shown in red) than in vgll3*LL individuals (11 genes, shown in green in Fig. 3a). Importantly, the *vgll3a* paralog showed reduced expression at the *Mature* stage across all comparisons. However, *yap1*, the primary competitor of *vgll3* in the Hippo pathway, displayed reduced expression at the *Mature* stage only in vgll3*EE. These results suggest an overall reduction in expression of Hippo pathway components and interacting partners during testicular maturation, with minor differences between the *vgll3* genotypes.

In addition, most of the GSI-correlated genes were the same genes that were differentially expressed between maturation stages in both genotypes, with only a few GSI-correlated genes not differentially expressed between the stages in each genotype: 6 genes in vgll3*EE and 18 genes in vgll3*LL (Supplementary Fig. 1c). This further suggests that most GSI-correlated genes exhibit maturation stage-specific expression. Interestingly, however, we found that the only gene with opposite correlation patterns between the genotypes, *lats1a*, was among the few genes not differentially expressed between the maturation stages (Supplementary Fig. 1d).

Next, we proceeded to investigate potential functional/molecular interactions among DE genes between the *Immature* and *Mature* stages that exhibited *vgll3* genotype-specific variation in expression (colored numbers in Fig. 3a). The predicted interactions between these genes revealed that from the *vgll3* genotype-specific comparisons, 7 and 19 DE genes within vgll3*LL and vgll3*EE individuals, respectively, were found to be connected in an interaction network (colored green and red in Fig. 3, b and c). Among the DE genes in vgll3*LL individuals, none had direct interactions with *vgll3* or *yap1*, and all showed higher expression at the *Immature* stage (Fig. 3e). Among the DE genes in vgll3*EE individuals, *greb1la*, *chmp2b*, *hif1a*, and *tead2* had direct interactions with *vgll3*, and all but 3 genes (*greb1la*, *chmp2b*, and *klf15a*) had direct interactions with *yap1*. Moreover, all of these genes except *rhoac* showed reduced expression at the *Immature* stage contrary to the DE genes in vgll3*LL. Among the DE genes in vgll3*LL individuals, none of them were upstream regulators of the Hippo pathway, whereas in vgll3*EE individuals, 5 key upstream regulators of the pathway were present (*frmd6a*, *sav1a*, *stk3b*, *tead2*, and *yap1*). Finally, none of the DE genes in vgll3*LL individuals formed an interacting hub, while several of hub genes were found among the DE genes in vgll3*EE such as *yap1*, *ppargc*, *rhoac*, *snaia*, and *hif1a*. In general, these findings indicate more Hippo-dependent changes in the testis of mature vs immature vgll3*EE individuals and a potential master regulatory role of *yap1* in this transition (Fig. 3c).

We observed paralog-specific expression patterns among the DE genes unique to both *vgll3* genotypes. For instance, while 2 paralogs of the *arhgap32* gene (*arhgap32d* and *arhgap32f*) showed increased expression at the *Mature* stage in both genotypes, 2 other paralogs, *arhgap32a* and *arhgap32c*, exhibited such increase only in the vgll3*LL genotype. This genotype-dependent divergence in paralog expression was also observed among other Hippo pathway interacting partners. However, most of these differences were present in vgll3*EE individuals, including *fgf14b* (vs *fgf14a*), *frmd6a* (vs *frmd6b/c*), *fstl3b* (vs *fstl3a*), *greb1lb* (vs *greb1la*), *kdm5ba/c/d* (vs *kdm5bb*), *klf15a/d* (vs *klf15b*), *myo10a* (vs *myo10b/c/d/f*), *rhoac* (vs *rhoaf/g/h*), *pcdh18a* (vs *pcdh18b/c*), *rxrbaa* (vs *rxrbab/c*), *sav1a* (vs *sav1b*), *stk3b* (vs *stk3a*), *tle1a* (vs *tle1b*), *tle4a* (vs *tle4b*), and *snaia* (vs *snaib*). In all these cases, the paralogs outside the parentheses exhibited genotype-specific expression divergence in vgll3*EE individuals. The direction of divergence in vgll3*EE was often toward reduced expression at the *Mature* stage and similar to the direction of the other paralogs that showed the same patterns but in both genotypes. *rhoac* and *sav1a* were 2 exceptions among them; *rhoac* showed increased expression at the *Mature* stage of vgll3*EE individuals, contrary to its paralogs (*rhoaf/g/h*). An opposite expression pattern was observed for *sav1a*, which displayed reduced expression at the *Mature* stage of vgll3*EE individuals, in contrast to *sav1b*, which had increased expression at the *Mature* stage in both genotypes. A more extreme example was paralogous expression divergence of the *prkar1a* gene: *prkar1ab* had higher expression only in vgll3*LL, while the other paralog, *prkar1ac*, showed lower expression only in vgll3*EE at the *Mature* stage. These observations suggest *vgll3* genotype-specific expression divergence in paralogs of the Hippo pathway-related genes during testicular maturation. In some cases, such as *prkar1a*, *rhoa*, and *sav1*, more pronounced divergence between genotypes may even indicate neofunctionalization between their paralogs.

### Genotype-specific gene coexpression networks

**Fig. 4.**
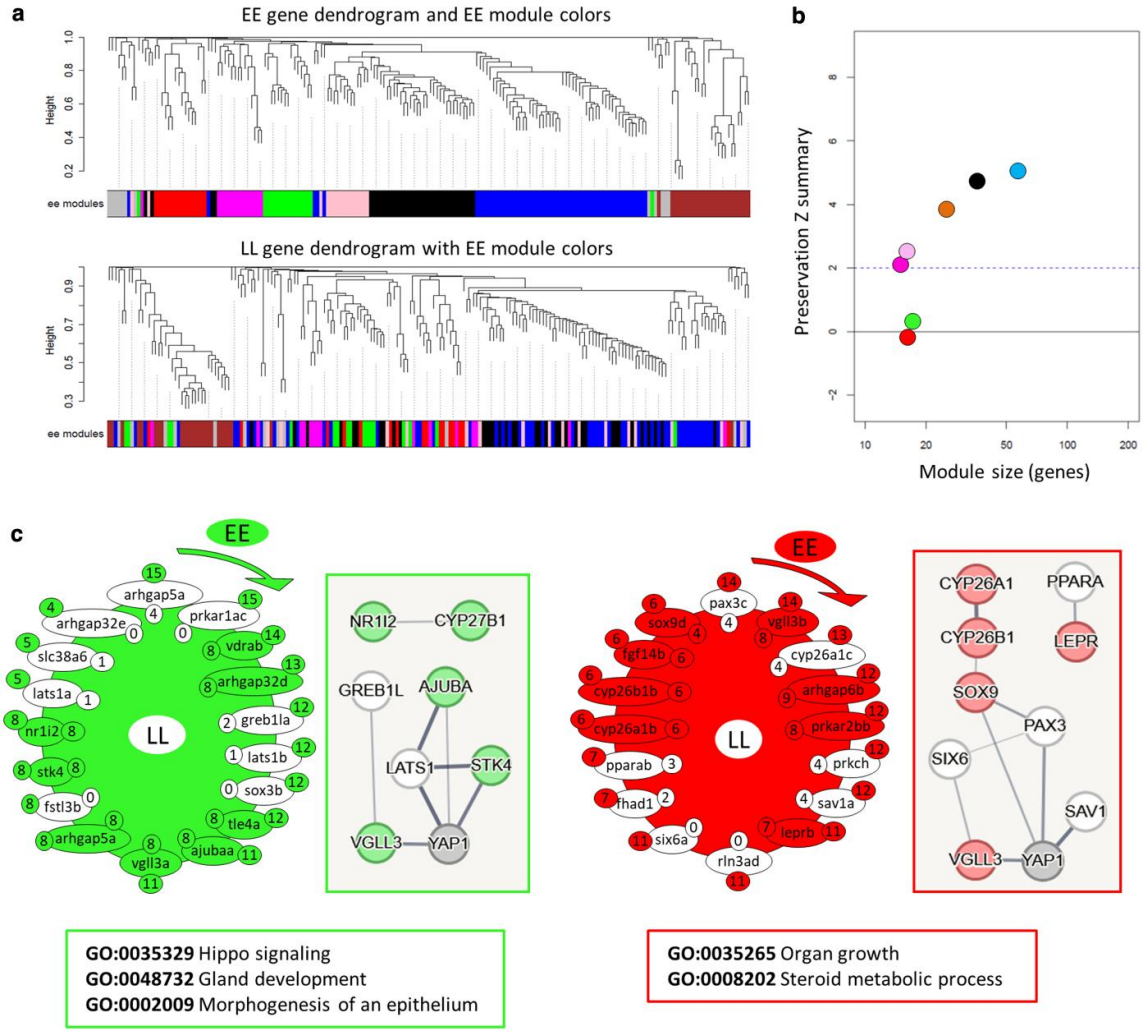
Coexpression analysis of vgll3*EE genotype in salmon testis. Dendrograms display clustering and preservation of vgll3*EE modules in vgll3*LL individuals, highlighting the lack of preservation in the top panels a). Zsummary scores indicate low preservation (<2) and moderate preservation (2 to 6) for vgll3*EE modules in vgll3*LL b). The genes in the least preserved vgll3*EE module in vgll3*LL individuals, along with their regulatory interaction maps and associated biological processes c). Nonpreserved genes are shown without color, and clockwise arrows indicate directions from the most to the least connected genes within the module, the numbers beside the genes and outside the module represent the number of expression correlations for each gene (with other genes within the same module) in vgll3*EE modules, and numbers inside represent the number of expression correlations for each gene preserved in vgll3*LL. Predicted interactions between the genes within each of the least preserved modules are depicted on the right side of the modules (via STRING). Line thickness is relative to interaction probabilities (thicker = higher). GO enrichment analysis of genes within each module is displayed below the modules, showing the associated biological processes (via WebGestalt).

To gain a better understanding of the transcriptional dynamics of Hippo pathway components and their interacting genes, we used network-based coexpression analyses. This approach enabled us to examine genotype-specific changes in each network. We found 7 modules in vgll3*EE (Fig. 4, a and b) of which 2 modules, green and red, showed low preservation (Zsummary < 1) in vgll3*LL; i.e. many of the genes in each module no longer had significant expression correlations in vgll3*LL genotype (the genes lacking color in Fig. 4c). In the red module, 8 out of 16 genes showed no coexpression preservation in vgll3*LL genotype individuals (Fig. 4c). Also in the green module, 5 out of 25 genes exhibited no coexpression preservation in vgll3*LL individuals (Fig. 4c). Using knowledge-based interactome prediction, we used the genes within each module to identify potential interactions among them. In both modules, at least 1 gene, among those lacking coexpression preservation, had a direct interaction with *vgll3* (i.e. *greb1la* in the green and *six6a* in the red module) (Fig. 4c). Interestingly, in each module, a different paralog of *vgll3* (*vgll3b* in the red and *vgll3a* in the green module) was found; however, both paralogs retained their coexpression preservation in vgll3*LL genotype individuals (Fig. 4c). Moreover, in each module, 1 upstream Hippo component encoding gene with direct interaction with *yap1* also lacked coexpression preservation in vgll3*LL genotype individuals (*lats1b* in the green and *sav1a* in the red module) (Fig. 4c). Furthermore, GO enrichment analysis of the red module in vgll3*EE revealed functional links to organ growth and steroid metabolism, whereas in the green module, the Hippo pathway itself, along with other GO terms related to gland development and epithelial morphogenesis, was enriched (Fig. 4c). This suggests that biological processes associated with these functions are among the most affected between the genotypes.

**Fig. 5.**
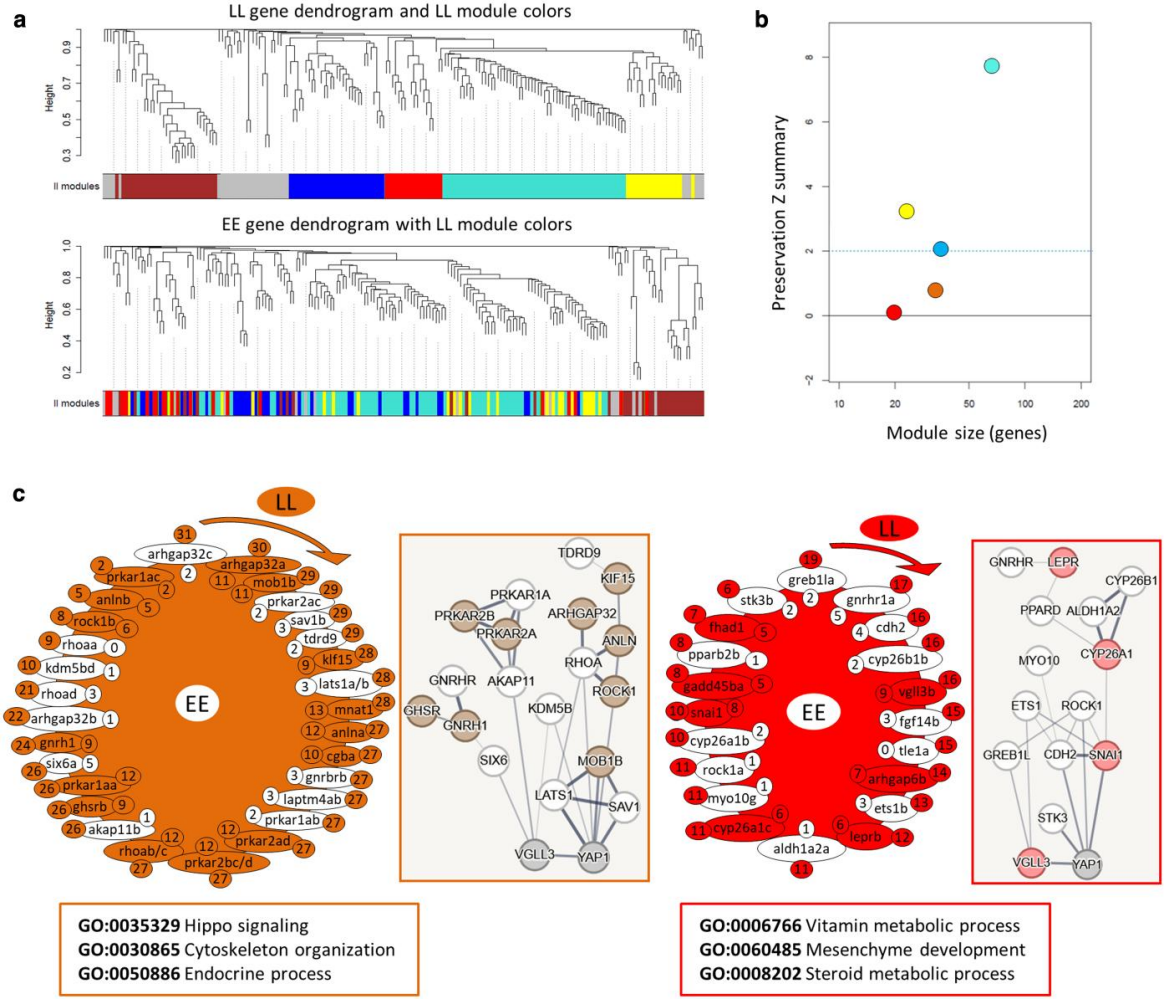
Coexpression analysis of vgll3*LL genotype in salmon testis. Dendrograms display clustering and preservation of vgll3*LL modules in vgll3*EE individuals, highlighting the lack of preservation in the top panels a). Zsummary scores indicate low preservation (<2) and moderate preservation (2 to 6) for vgll3*LL modules in vgll3*EE b). The genes in the least preserved vgll3*LL module in vgll3*EE, along with their regulatory interaction maps and associated biological processes c). Nonpreserved genes are shown without color, and clockwise arrows indicate directions from the most to the least connected genes within the module, the numbers beside the genes and outside the module represent the number of expression correlations for each gene (with other genes within the same module) in vgll3*LL modules and numbers inside represent the number of expression correlations for each gene preserved in vgll3*EE. Predicted interactions between the genes within each of the least preserved modules are depicted on the right side of the modules (via STRING). Line thickness is relative to interaction probabilities; GO enrichment analysis of genes within each module is displayed below the modules, showing the associated BPs (via WebGestalt).

In vgll3*LL compared to vgll3*EE individuals, we identified 5 modules, 2 of which (red and brown) showed low preservation levels (Zsummary < 1) (Fig. 5, a and b). Within the red module, interaction predictions revealed 2 genes, *ets1b* and *greb1la*, that directly interact with *vgll3*. Both genes lost their coexpression preservation in vgll3*EE individuals (Fig. 5c). Interestingly, *greb1la* was also the most connected gene in this module, showing significant expression correlation with 19 out of 20 genes. Moreover, *stk3b*, an upstream regulator of the Hippo pathway with a direct interaction with *yap1*, also lacked expression correlation in vgll3*EE individuals. In the brown module, 3 genes, *six6a*, *akap11b*, and *kdm5bd*, had direct interactions with *vgll3* and exhibited a loss of coexpression with other module genes in vgll3*EE individuals (Fig. 5c). Similarly, 3 upstream regulators of the Hippo pathway, *lats1a*, *lats1b*, and *sav1b*, which directly interact with *yap1*, also lacked expression correlation in vgll3*EE individuals. Finally, GO enrichment analysis of the vgll3*LL modules showed that genes in the red module were again associated with steroid metabolism, similar to the red module in vgll3*EE, but also linked to processes such as mesenchyme development and vitamin metabolism (Fig. 5c). In the brown module, the Hippo pathway was again enriched, this time alongside GO terms related to endocrine processes and cytoskeletal organization. These results further highlight key biological processes likely affected between the genotypes, with the Hippo pathway and steroid metabolism emerging as shared components in both.

## Discussion

Sexual maturation, or puberty, is initiated in the brain, primarily when the hypothalamus secretes gonadotropin-releasing hormone (GnRH), which triggers the pituitary gland to release luteinizing hormone (LH) and follicle-stimulating hormone (FSH) (Herbison 2016). The activation of the FSH signal initiates a cascade of signaling pathways that regulate various metabolic, developmental, and cellular processes essential for spermatogenesis (Ni *et al.* 2019, 2020). Among these pathways, the Hippo pathway has recently been identified as a signal directly regulated by FSH, playing significant roles not only in the early stages of testicular maturation but also in later stages of spermatogenesis and related pathologies (Abou Nader et al. 2019; Sen Sharma et al. 2019; Zhang et al. 2019; Meng et al. 2020; Yu et al. 2021; Onel et al. 2023). However, compared to other well-known pathways involved in this process, such as the cAMP-PKA, ERK1/2-MAPK, PI3K-AKT, and TGF-β signaling pathways (Ni *et al.* 2019, 2020), much less is understood about the specific molecular players and their functions within the Hippo pathway cascade. Previous research in Atlantic salmon suggests that the *vgll3* genotype influences Hippo signaling and lipid metabolism in adipose tissue, potentially initiating cross-talk with the HPG axis to coordinate maturation onset (Ahi et al. 2024b, 2025c). Differential expression of *vgll3* and Hippo-related genes has also been observed in the brain and pituitary prior to testis activation (Ahi et al. 2022, 2023, 2025d; Huang et al. 2025). Similar patterns in other vertebrates show that Hippo signaling in adipose tissue can influence HPG axis activity through YAP/TAZ pathways (Choi et al. 2024; Ahi et al. 2025b) and regulate Sertoli cell function and spermatogenesis in the testis (Levasseur et al. 2017; Kjærner-Semb et al. 2018; Lalonde-Larue et al. 2022). In this study, we explored how different genotypes of the *vgll3* gene impact transcriptional activity of the Hippo pathway components in immature and mature testis. We also examined whether *vgll3*-associated transcriptional changes in the testis could serve as early indicators of maturation, even before any visible signs of maturation become apparent in the testis. The molecular signaling regulated by the Hippo pathway is known to play essential and conserved roles from early stages of testicular development to final stages of spermatogenesis (Sun et al. 2008 ; Abou Nader et al. 2019; Sen Sharma et al. 2019; Onel et al. 2023). This allowed us to gain insights into how Hippo pathway signaling is regulated in the testes of Atlantic salmon, which have a reproductive strategy closely linked to seasonal variations and body fat reserves.

### Potential heterochrony in the Hippo-dependent regulation of early signals of spermatogenesis

The expression patterns of Hippo pathway components and their interacting partners across the 3 time points suggest a potential heterochronic shift in the timing of testicular development and spermatogenesis between the *vgll3* genotypes, with vgll3*EE individuals displaying more advanced patterns than vgll3*LL individuals. For example, at the earliest immature time point (spring: *Immature 1*), we found the expression of genes encoding Hippo components/interacting partners with an enhancing role in early stages of spermatogenesis to be induced in the testis of vgll3*EE individuals. Notably, we found that *stk3a*, a paralog of a gene encoding a key kinase in the Hippo pathway (also known as *MST2*), is more highly expressed in the testis of individuals with the vgll3*EE than the vgll3*LL genotype at the earliest *Immature* stage (spring). In mice, MST2 activation is required for YAP suppression, which promotes the differentiation of the epididymal initial segment (IS) in the testis (Meng et al. 2020). The differentiation of the epididymal IS is considered an early sign of developmental changes in the testis, leading to the onset of testicular maturation and the increase in androgen (testosterone) levels (O’Hara et al. 2011). Consistently, we also found induction of a *KLF15* paralog gene (*klf15b*) in the vgll3*EE genotype, which is known as a direct target of androgen in Sertoli cells in mice (de Gendt et al. 2014).

The observation that components of similar molecular signals involved in the early stages of testicular development and spermatogenesis showed increased expression in the vgll3*LL genotype at the later immature time point suggests a potential heterochrony between the genotypes in the timing of these developmental processes. For example, increased expression of genes related to the ROCK/LIMK signaling pathway was observed in both genotypes, but involving different genes and time points. In vgll3*EE, *rock2b* and *pparac* were upregulated during the early immature stage, while in vgll3*LL males, *limk2b* and *rhoae* showed increased expression at the later immature stage. All 4 genes are functionally linked to early spermatogenesis; e.g. *rock2b*, *limk2b*, and *rhoae* contribute to the formation of cell–cell junctions between Sertoli cells and spermatogonia (Takahashi et al. 2002; Lui et al. 2003a, 2003b), while *pparac*, a paralog of *PPARα*, regulates lipid metabolism and energy availability in the testis (Monrose et al. 2021). The upregulation of *pparac* in vgll3*EE males may reflect either early activation by FSH, as supported by our prior observation of early *fshb* expression in the pituitary (Ahi et al. 2022, 2023) and known FSH-induced expression of *PPARα* and *MST2* in mammals (Schultz et al. 1999; Sen Sharma et al. 2019), or higher lipid availability in this genotype (Hasan et al. 2020; Ahi et al. 2024b). Interestingly, another paralog of the *KLF15* gene (*klf15d*) was found to have higher expression in the vgll3*LL genotype at this time point, suggesting a subsequent heterochronic shift in androgen effects on Sertoli cells in this genotype (de Gendt et al. 2014). However, more developmental time points are needed to obtain a clearer and more detailed understanding of these molecular cascades during testicular maturation in both genotypes.

At the mature time point, heterochrony appears to be maintained, with genes involved in various stages of testicular development and spermatogenesis showing shifts, as suggested by early-stage genes being more highly expressed in vgll3*LL individuals, while later-stage markers have increased expression in vgll3*EE individuals. For instance, *kdm5b* (also known as *PLU-1* or *JARI1B*), a gene with essential role in premeiotic spermatogonial proliferation and repression of terminal spermatocyte differentiation (Simpson et al. 2005), had higher expression in the testis of vgll3*LL individuals. Other genes with essential role in earlier stages of spermatogenesis that had higher expression in vgll3*LL individuals include *tdrd9* and *ppm1a* (or *PP2AC*), both required for the initiation of meiotic phase (Shoji et al. 2009; Chen et al. 2022), and *prkar1* with critical functions during the early prophase I stage of meiosis (the pachytene stage) (Burton et al. 2006). In contrast, genes involved in the later stages of spermatogenesis had higher expression in the mature testis of vgll3*EE individuals such as *arhgap6*, required for actin remodeling and sperm motility (Almog et al. 2008); *aldh1a2*, highly expressed during spermatocyte differentiation and spermatid maturation (Griswold et al. 2022); and *ppara*, required for lipid metabolism and providing energy for sperm motility (Froment et al. 2006). It is important to note that these spermatogenesis-related genes are expressed in both genotypes; the observed differences reflect relative expression levels rather than presence or absence. This likely reflects variation in the cellular composition of the mature testis tissue at the time of sampling, with vgll3*EE males having a higher proportion of germ cells at later stages of spermatogenesis, and vgll3*LL males enriched for cells at meiotic or immediately postmeiotic stages. Such a shift in relative expression supports the hypothesis of heterochrony in spermatogenic progression, not a fundamental difference in gene activity between genotypes.

### Paralog-specific expression patterns during spermatogenesis

A paralog of an early meiosis stage marker, *prkar1ab*, exhibited higher expression in vgll3*LL, while the other paralog, *prkar1ac*, showed lower expression in vgll3*EE during the *Mature* stage. This indicates that the testis of vgll3*LL individuals is more active at earlier stages of spermatogenesis (Burton et al. 2006). Such paralog specificity followed the same trend of heterochrony, i.e. earlier-stage genes having higher expression in vgll3*LL at each time point (and vice versa).For instance, we observed the reduced expression of genes known to be more active in earlier stages of spermatogenesis in the testis of vgll3*EE individuals during the *Mature* stage. Among these were 3 *kdm5b* paralogs (*kdm5ba/c/d*), which act as inducers of spermatogonial proliferation and repressors of terminal spermatocyte differentiation (Simpson et al. 2005); 2 *klf15* paralogs (*klf15a/d*), markers of early-stage androgen sensing in Sertoli cells (de Gendt et al. 2014); *myo10a* paralog, a marker of midspermatogenesis stages and spindle formation during meiosis (Brieño-Enríquez et al. 2017); *rhoac* paralog, a marker of cell junctions in spermatogonia (Lui et al. 2003a, 2003b); *stk3b* paralog, a marker of earlier stages of testicular development (Meng et al. 2020); *pcdh18a* paralog, a marker of the premeiotic stage of spermatogenesis (Jan et al. 2017); and *tle4a* and *snaia* paralogs, markers of spermatogonial differentiation (Micati et al. 2018; Zhang et al. 2021). These paralog-specific patterns align with earlier findings, which showed that markers of earlier stages of spermatogenesis are more active in the testis of vgll3*LL individuals. Moreover, these expression differences between the paralogs suggest potential subfunctionalization among these paralogs, which appears to be influenced by distinct Hippo pathway activity. An interesting example is the difference between the *lats1* gene paralogs, key components of the Hippo pathway recently shown to play an important role in testicular maturation and growth in mice (Abou Nader et al. 2022, 2024). While the expression of both *lats1a* and *lats1b* showed positive correlation with testicular growth (GSI) in the vgll3*LL genotype, only *lats1a* showed a significant correlation in vgll3*EE, remarkably, in the opposite direction (negative correlation). This may indicate a paralog-specific function of *lats1a* between the genotypes during testicular development in Atlantic salmon.

### Different Hippo pathway components and interacting partners may participate in the testicular maturation shift

By combining coexpression analysis and interactome prediction, we identified coexpression modules most affected by differences between the *vgll3* genotypes. This approach also allowed us to pinpoint potential key contributors to the distinct gene expression patterns observed between the genotypes. Importantly, within the most affected gene coexpression networks (the least preserved), we identified distinct *vgll3* paralogs. While the coexpression of these paralogs remained unaffected, several key interacting partners and Hippo pathway components appeared to be differentially affected between genotypes. Among the affected upstream components of the Hippo pathway were *lats1*, *sav1a*, and *stk3b*, which have been previously discussed for their roles in testicular development and spermatogenesis (see above). Downstream effectors of the Hippo pathway showing direct connections with *vgll3* included *kdm5bd* (as described above) and *ets1b*. Recent research indicates that Vgll3 can directly bind to regulatory sequences of the *ets1* gene in salmon testes (Verta et al. 2024). ETS1, a transcription factor, has been implicated in mammalian spermatogonia stem cell differentiation (Lu et al. 2024) and is also present in mature spermatozoa (Pittoggi et al. 2001). Furthermore, ETS1 binding sites have been identified in enhancers driving mitotic (but not meiotic) spermatogenesis in mammals (Maezawa et al. 2020). In our study, we found a genotype-specific expression correlation between *ets1b* and *vgll3b* paralog, observed only in the vgll3*LL genotype. This may suggest differences in the initiation of spermatogonial proliferation and differentiation between genotypes. Moreover, we identified 3 genes, *greb1la*, *six6a*, and *akap11b*, that directly interact with *vgll3* but are not classified as Hippo pathway components. These genes are implicated in life history traits in salmonids. For instance, *greb1l* has been associated with migration timing in Chinook salmon and steelhead (Collins et al. 2020; Koch and Narum 2020; Horn and Narum 2023), while *six6* and *akap11* have been linked to age at maturity in Atlantic salmon (Barson et al. 2015; Kurko et al. 2020; Moustakas-Verho et al. 2020; Sinclair-Waters et al. 2020; Ahi et al. 2024a; Frapin et al. 2025). In mice, the antisense transcript of *SIX6* (*SIX6OS1*) is required for testicular development and spermatogenesis (Gómez-H et al. 2016), but the specific role of *SIX6* in testicular function remains unexplored in vertebrates. Similarly, *GREB1L* has recently been proposed as a marker for male infertility in mammals (Lillepea et al. 2024), and its paralog *GREB1* is involved in estrogen-dependent functions of Sertoli cells (Lin et al. 2014). However, the precise role of *greb1l* in vertebrate spermatogenesis remains unclear. Lastly, *AKAP11* (also known as *hAKAP220*) is expressed throughout spermatogenesis and is believed to play a role in cytoskeletal structure formation (Reinton et al. 2000). These findings emphasize the significance of *vgll3* in testicular maturation, extending beyond its conventional role as a cofactor of the Hippo pathway. As suggested, *vgll3* may influence testicular processes by interacting with other signaling pathways (Verta et al. 2024).

Finally, functional GO enrichment of the least preserved modules revealed *vgll3*-associated biological processes that may regulate the timing of testis maturation. For instance, in both *vgll3* genotypes, the red module genes were enriched for steroid metabolic processes. Steroid hormones are essential for initiating testis growth and germ cell differentiation (Flück and Pandey 2014; Li et al. 2024). Strikingly, differential timing in the regulation of steroidogenic genes has recently been implicated as a potential mechanism underlying cross-species heterochrony in testis maturation in mammals (Wang et al. 2024). Furthermore, in both red modules, we found coexpression of *leprb*, a receptor of the leptin signaling pathway. Leptin is a well-established link between nutritional status and gonadal development (Hileman et al. 2000; Sengupta et al. 2019; Tsakoumis et al. 2022), and it has been shown to influence both steroidogenesis (Martins et al. 2017) and the timing of puberty onset (Gueorguiev et al. 2001). Importantly, the Hippo pathway has recently emerged as a signaling hub with direct regulatory cross-talk with leptin signaling in environmental contexts (Ahi et al. 2025b), and it also functions as a potent regulator of steroidogenesis (Shin et al. 2020; Mizutani et al. 2023).

### Conclusion

This study offers molecular evidence that connects testicular gene expression with genotype-related variation in life history strategies, particularly related to sexual maturation. Our results provide evidence of gene expression that supports the ability to predict early maturation in the testis before visible changes linked to maturation manifest in immature male Atlantic salmon. This predictability is influenced by a unique transcriptional signature involving components and interacting partners of the Hippo pathway, which appears significantly affected in individuals with both *early* and *late vgll3* genotypes. Moreover, at each compared stage, the testis of individuals with *early vgll3* genotype seems to have a transcriptional signature suggestive of more advanced stages of spermatogenesis, indicating potential heterochronic shift. These findings may imply that particular elements within the Hippo signaling pathway as well as less known factors with potential interactions with the pathway (e.g. *greb1lb* and *six6a*) could play a substantial role in driving the varying effects of *vgll3* genotypes on testicular gene expression and the timing of maturation. Moreover, given that the Hippo pathway is an environmental sensing pathway, particularly responsive to food availability and temperature, both of which influence maturation, these findings suggest a likely link between the environment and heterochronic events through the *vgll3*/Hippo axis, leading to differences in age at maturity. However, further functional assessments are needed to confirm these proposed distinctions.

## Supplementary Material

jkaf196_Supplementary_Data

## Data Availability

All the gene expression data generated during this study are included in this article as supplementary file. The gene expression data have also been deposited in the GEO database at NCBI under accession number GSE303890. The custom-made NanoString panel used in this study is available to order via the nCounter platform (nanostring.com/products/ncounter-assays-panels) with codeset quote number SQ-39593, under the name salmon-2. Supplemental material available at *G3* online.
