# Supplementary material for: Testicular heterochrony in vgll3-mediated maturation age in Atlantic salmon
Source: G3 (Bethesda). 2025 Aug 21;15(11):jkaf196. doi: 10.1093/g3journal/jkaf196 (PMC12609175; doi:10.1093/g3journal/jkaf196)
Supplement: jkaf196_Supplementary_Data [file jkaf196_supplementary_data.zip › Supplementary_Figure_1_G3-2025-406144.docx]

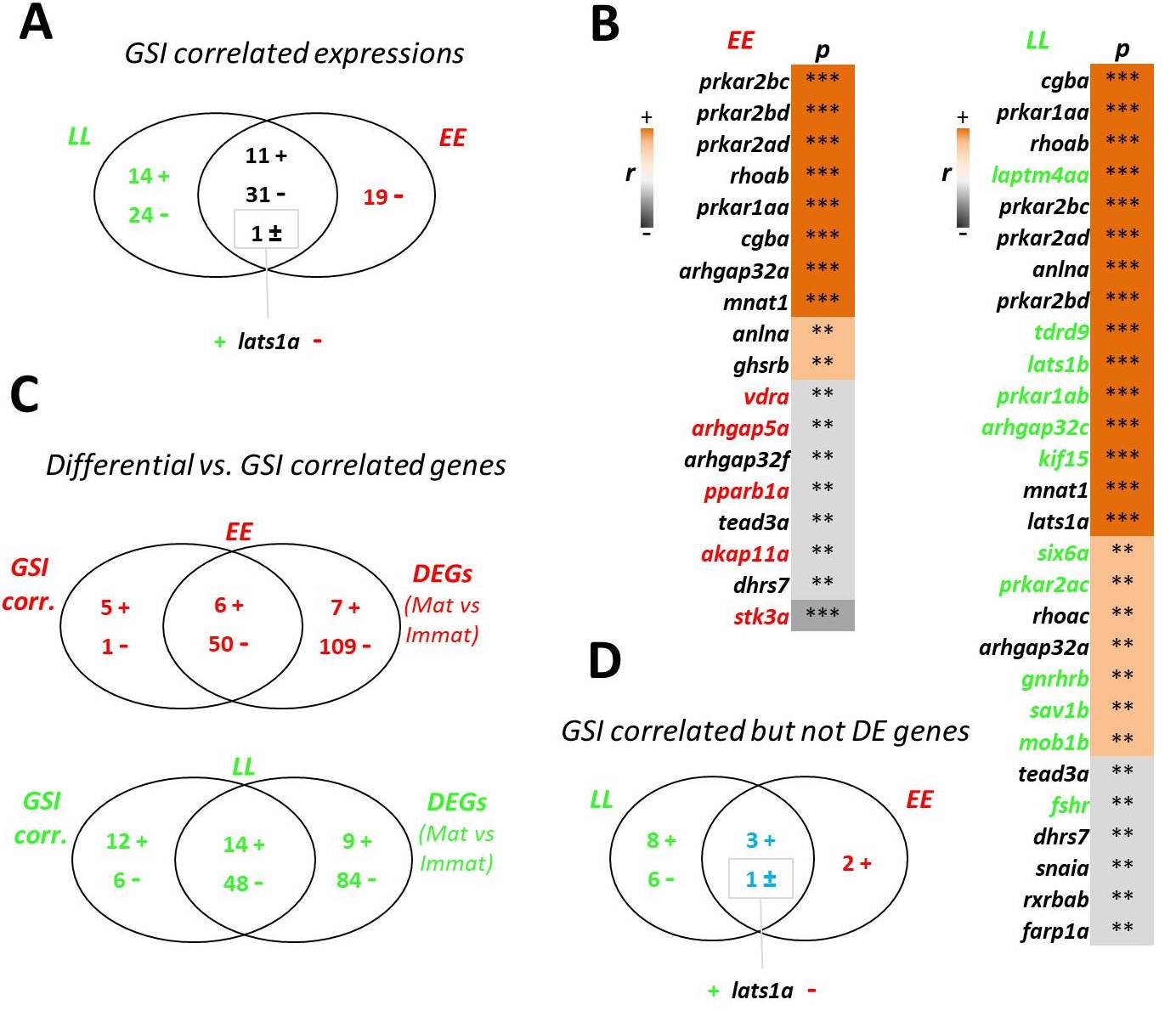


**Supplementary Figure 1.** **Genes showing expression patterns correlated with GSI in the testis.** (**A**) The Venn diagram represents the numbers of genes significantly correlated with GSI that are unique to each of the *vgll3* genotypes or shared between them. (**B**) The most significant Pearson correlations between gene expression and GSI in the testis. EE and LL indicate *vgll3*EE* and *vgll3*LL* genotypes, respectively, and *p* and *r* indicate p-values (** < 0.01; *** < 0.001) and Pearson correlation coefficient. Gene colors correspond to the color codes within the A Venn diagram. (**C**) The Venn diagrams show the number of genes significantly correlated with GSI and/or differentially expressed between maturation stages within each genotype. (**D**) The Venn diagrams show the number of genes significantly correlated with GSI but not differentially expressed, either unique to each genotype or shared between them.
