# Supplementary material for: Testicular heterochrony in vgll3-mediated maturation age in Atlantic salmon
Source: G3 (Bethesda). 2025 Aug 21;15(11):jkaf196. doi: 10.1093/g3journal/jkaf196 (PMC12609175; doi:10.1093/g3journal/jkaf196)
Supplement: jkaf196_Supplementary_Data [file jkaf196_supplementary_data.zip › Supplementary_File_Legends_G3-2025-406144.docx]

**Supplementary File Legends**

**Supplementary File 1.** The information about the gene probes, expression data and quality control

**Supplementary File 2.** STRING input and statistical analysis
